# The Sensor Proteins BcSho1 and BcSln1 Are Involved in, Though Not Essential to, Vegetative Differentiation, Pathogenicity and Osmotic Stress Tolerance in *Botrytis cinerea*

**DOI:** 10.3389/fmicb.2019.00328

**Published:** 2019-02-25

**Authors:** Weichao Ren, Na Liu, Yalan Yang, Qianqian Yang, Changjun Chen, Qingli Gao

**Affiliations:** ^1^College of Plant Protection, Nanjing Agricultural University, Nanjing, China; ^2^Institute of Biotechnology, Zhejiang University, Hangzhou, China; ^3^College of Plant Health and Medicine, Qingdao Agricultural University, Qingdao, China; ^4^Plant Protection Station of Pizhou City, Xuzhou, China

**Keywords:** high-osmolarity glycerol pathway, osmosensor, development, virulence, osmotic stress

## Abstract

High-osmolarity glycerol (HOG) signaling pathway belongs to mitogen-activated protein kinase (MAPK) cascades that regulate responses of organism to diverse extracellular stimuli. The membrane spanning proteins Sho1 and Sln1 serve as biosensors of HOG pathway in *Saccharomyces cerevisiae*. In this study, we investigated the biological functions of *BcSHO1* and *BcSLN1* in the gray mold fungus *Botrytis cinerea*. Target gene deletion demonstrated that both *BcSHO1* and *BcSLN1* are important for mycelial growth, conidiation and sclerotial formation. The *BcSHO1* and *BcSLN1* double deletion mutant Δ*BcSln1-Sho1* produced much more, but smaller sclerotia than Δ*BcSho1* and the wild-type (WT) strain, while Δ*BcSln1* failed to develop sclerotia on all tested media, instead, formed a large number of conidia. Infection tests revealed that the virulence of Δ*BcSln1-Sho1* decreased significantly, however, Δ*BcSho1* or Δ*BcSln1* showed no difference with the WT strain. In addition, Δ*BcSln1-Sho1* exhibited resistance to osmotic stress by negatively regulating the phosphorylation of BcSak1 (yeast Hog1). All the phenotypic defects of mutants were recovered by target gene complementation. These results suggest that *BcSHO1* and *BcSLN1* share some functional redundancy in the regulation of fungal development, pathogenesis and osmotic stress response in *B. cinerea*.

## Introduction

*Botrytis cinerea* (teleomorph: *Botryotinia fuckeliana*) is a devastating fungal pathogen that causes gray mold on over 500 plant species worldwide (Fillinger and Elad, 2016). Moreover, *B. cinerea* infects crop hosts during both pre- and post-harvesting phases and leads to huge economic losses (Dean et al., 2012). Currently, because of the lack of resistant varieties, chemical control is still the most effective strategy for controlling gray mold, however, many kinds of fungicides have lost effectiveness due to resistance development (Hu et al., 2016; Weber and Hahn, 2019). Therefore, exploring the molecular mechanisms underlying vegetative differentiation, pathogenesis and stress response in *B. cinerea* will contribute to establish more efficient disease management strategies.

**FIGURE 1:**
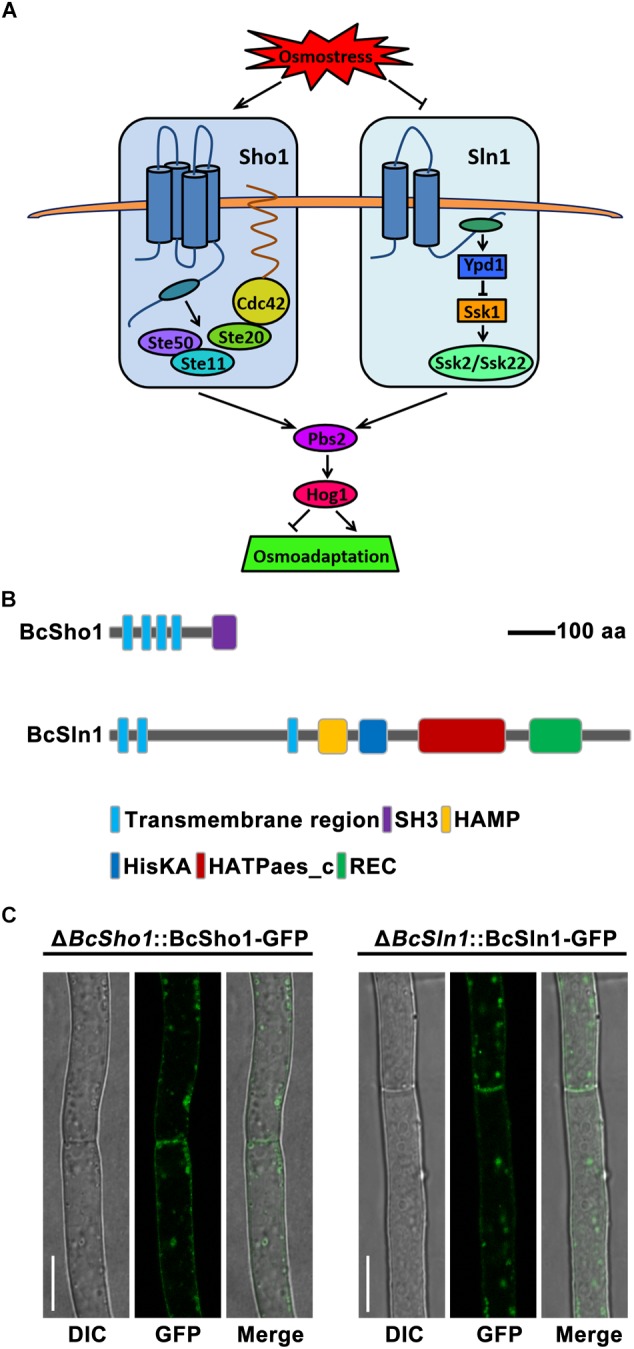
Model of the yeast hyperosmotic-response MAPK pathway. **(A)** The osmotic signals from Sho1 or Sln1 are transduced by unique components and converge to activate Pbs2. The Sho1 branch requires Cdc42, Ste20, and Ste50 to activate Ste11. The Sln1 protein activates Ssk2 and Ssk22 through Ypd1 and Ssk1. Any of Ste11, Ssk2, or Ssk22 is able to activate Pbs2, which then phosphorylates Hog1, resulting in translocation of Hog1 to the nucleus that regulates responsible genes expression for osmoadaptation. **(B)** The schematic diagram of Sho1 and Sln1 orthologs in *Botrytis cinerea*. The functional domains were retrieved in EnsemblFungi (https://fungi.ensembl.org/Botrytis_cinerea/Info/Index). **(C)** Subcellular localization of BcSho1-GFP and BcSln1-GFP fusion proteins in *B. cienrea*. Bars, 10 μm.

Cells sense and respond to a variety of extracellular signals through ubiquitous mitogen-activated protein kinase (MAPK) cascades (Pearson et al., 2001). To date, five MAPK pathways have been identified in the eukaryotic model *Saccharomyces cerevisiae*, which regulate diverse physiological processes, including filamentation, sporulation, osmoregulation, cell wall integrity and pheromone response (Gustin et al., 1998). Osmoregulation is mainly controlled by the (high-osmolarity glycerol) HOG signaling pathway to adapt external hyperosmotic stress (Hohmann et al., 2007). Specifically, signals from the Sho1 and Sln1 branches are independently transduced by unique components and converge to activate the MAPK kinase (MAPKK) Pbs2. Cdc42, Ste20, and Ste50 are essential for Sho1 branch to activate the MAPK kinase kinase (MAPKKK) Ste11 (Saito and Tatebayashi, 2004). The Sln1 branch activates two partially redundant MAPKKs, Ssk2, and Ssk22 through Ypd1 and Ssk1 to Reiser et al. (2003), Pbs2 could be activated by any of the three osmoresponsive MAPKKKs (Ste11, Ssk2, or Ssk22), then the activated Pbs2 phosphorylates the MAPK Hog1, resulting in the translocation of Hog1 to nucleus and finally regulates the expression of responsible genes (Figure 1A; O’Rourke and Herskowitz, 2003).

The HOG pathway also involved in oxidative and cell wall stress response, through the interaction with the cell wall integrity (CWI) pathway (Brewster et al., 1993; Schüller et al., 1994). In addition to the conserved function of osmoregulation, the HOG pathway also plays several species-specific roles (Jiang et al., 2018). For example, the HOG pathway is dispensable for pathogenesis in some pathogenic fungi, such as *Magnaporthe oryzae*, *Bipolaris oryzae*, and *Colletotrichum orbiculare* (Li et al., 2012; Turrà et al., 2014), however, mutants blocked in this pathway exhibit severe defect in host infection in *Candida albicans*, *Fusarium graminearum*, *Zymoseptoria tritici*, *Verticillium dahlia*, and *Cochliobolus sativus* (Jiang et al., 2018). In phytopathogenic fungi, the HOG pathway also plays species-specific roles in growth and development, such as the regulation of conidiation and perithecium formation in *F. graminearum* and microsclerotium formation in *V. dahliae* (Zheng et al., 2012; Wang et al., 2016). Nevertheless, in general, the HOG pathway play crucial roles in oxidative stress responses and tolerance to phenylpyrrole and dicarboximide fungicides, despite its function in response to cell wall and other stresses may vary among different fungi (Jiang et al., 2018).

There are several core elements of HOG pathway in *B. cinerea*, including the osmosensor histidine kinase BcOs1, the histidine phosphotransfer protein BcHpt1, two response regulators BcRrg1 and BcSkn7, the MAPKKK BcOs4, the MAPKK BcOs5 and the MAPK BcSak1 (Viefhues et al., 2015). Up to now, most of the core proteins have been well characterized and showed that the HOG pathway plays unique roles in vegetative differentiation and stress responses in *B. cinerea* (Viaud et al., 2006; Segmüller et al., 2007; Liu et al., 2008; Yan et al., 2010; Yang et al., 2012). To further understand the function of HOG pathway in *B. cinerea*, we identified and investigated the upstream sensor proteins BcSho1 and BcSln1 in this pathway, and demonstrated the roles in fungal development, virulence and stress responses.

## Materials and Methods

### Fungal Strains and Culture Conditions

Strain B05.10 of *B. cinerea* was used as a recipient strain for the transformation experiments and as a wild-type control. The WT, resultant gene deletion and complemented strains were grown at 25 C on potato dextrose agar (PDA), complete medium (CM) and minimal medium (MM) for mycelial growth tests (Ren et al., 2018). *B. cinerea* strains were incubated on PDA plates under white light for conidiation or in the darkness for sclerotial formation. Sensitivity tests to osmotic stress were performed on PDA plates containing NaCl (1.5 M), KCl (1.5 M) or Sorbitol (2 M). The inhibition ratio of mycelial growth was calculated as a percentage of colony radial growth on medium with inhibitor compared with that on normal medium. Each experiment was repeated three times independently.

### Gene Deletion and Complementation

Generation of the gene deletion and complementation strains were performed with the protocol described previously (Ren et al., 2017). To obtain *BcSHO1* and *BcSLN1* double deletion mutant, *BcSHO1* was knocked out from *BcSLN1* single deletion mutant. The primers used to amplify gene fragments were listed in Supplementary Table S1. Putative gene deletion mutants were identified by PCR and further confirmed by southern blotting analyses.

### Construction of GFP Fusion Cassettes and Microscopy

To construct BcSho1-GFP fusion cassette, the open-reading fragment (without stop codon) of *BcSHO1* was amplified and assembled with the NcoI-digested plasmid pNAN-OGG (Schumacher, 2012) using a One Step Cloning Kit (Vazyme Biotech, Nanjing, China). Using the same strategy, BcSln1-GFP cassette was also constructed. The resulting recombinant vectors were sequenced to ensure accuracy of the in-frame fusion region, and then transformed into the corresponding deletion mutants. The resultant transformants were screened by PCR and fluorescence signal. Subcellular localization was observed with a confocal laser scanning microscope (Leica TCS SP8, Germany).

### Pathogenicity Assays

Primary leaves of strawberry were point-inoculated with the mycelial plugs of 3-day-old cultures. Prior to inoculation, the leaves were wounded with a sterilized needle to facilitate the penetration of plant tissues. Apple and grape fruits were harvested at the maturity stage, and were also wounded with a sterilized needle before inoculation. The detached host samples were moisturized and incubated at 25 C with 16 h of daylight. Lesion diameters were measured at 3 days (leaves) or 4 days (fruits) post-incubation using the crossing method. Three samples were incubated for each strain, and the experiments were repeated three times independently.

### Sensitivity Tests to Different Fungicides

Mycelial plugs were taken from the colony margin and placed on the center of PDA plates amended with fludioxonil (0, 0.00625, 0.0125, 0.025, 0.05, and 0.1 μg/ml) or procymidone (0, 0.0625, 0.125, 0.25, 0.5, and 1 μg/ml) or iprodione (0, 0.125, 0.25, 0.5, 1, and 2 μg/ml). Three replicates per concentration for each strain. After incubation for 4 days at 25 C, the colony diameter was measured perpendicularly. EC_50_ (the effective concentration for 50% inhibition of mycelial growth) was calculated by the regression of the probit of the percentage of inhibition of radial growth against the logarithmic value of fungicide concentration. The experiments were repeated three times independently.

### Determination of Intracellular Glycerol Content

Strains were grown in liquid YEPD (1% yeast extract, 2% peptone, and 2% dextrose) for 48 h and then treated with NaCl (0.8 M) for 30 min. The mycelia were harvested and ground in liquid nitrogen. 100 mg of mycelial powder were added to 1 ml of glycerol extraction buffer (Applygen Technologies Inc., Beijing, China), homogenized and centrifuged at 5000 g at for 20 min at 4 C. 5 μl supernatant of each sample was mixed with 195 μl of glycerol detection buffer (Applygen Technologies Inc.) in 96-well plate. The glycerol concentration was determined using an automated microplate reader (Bio-Rad, Japan) at 550 nm after the mixture had been incubated at 37 C for 20 min. The experiments were repeated three times independently.

### Protein Manipulation and Western Blotting

Protein extraction was performed as described previously (Yu et al., 2014). The total proteins were separated by 12% sodium dodecyl sulfate-polyacrylamide gel electrophoresis (SDS-PAGE) and transferred to Immobilon-P transfer membrane (Millipore, Billerica, MA, United States). Phosphorylation of BcSak1 was detected by using an antibody against dually phosphorylated p38 (Thr180/Tyr182) (Cell Signaling Technology, Beverly, MA, United States). In addition, the samples were detected with anti-Hog1 antibody (C-terminal anti-Hog1) (Santa Cruz Biotechnology, Santa Cruz, CA, United States) as a reference. The experiments were repeated three times independently.

## Results

### Identification of *BcSHO1* and *BcSLN1* in *B. cinerea*

The Sho1 and Sln1 orthologs of *B. cinerea* were identified by BLASTP search of the *B. cinerea* genome database^1^ using *S. cerevisiae* Sho1 and Sln1 as queries, respectively. *BcSHO1* (Gene ID: Bcin08p05150) is predicted to encode a 312 amino-acid protein with 32% identity to the transmembrane osmosensor Sho1 of *S. cerevisiae*. Protein domain analysis by SMART^2^ revealed that BcSho1 contains four characteristic transmembrane domains and a C-terminal SH3 domain. *BcSLN1* (Gene ID: Bcin14p01870) is predicted to encode an 1184 amino-acid protein with 38% identity to the transmembrane histidine kinase and osmosensor Sln1 of *S. cerevisiae*. BcSln1 contains three characteristic transmembrane domains, a SH3 domain, a HAMP domain, a HisKA domain, a HATPase-c domain and a REC domain (Figure 1B). Subcellular localization showed that BcSho1-GFP and BcSln1-GFP fusion proteins were mainly located close to the cell membrane of mycelia in *B. cinerea* in some type of vesicular patches (Figure 1C).

### Deletion of *BcSHO1* and *BcSLN1* in *B. cinerea*

To investigate the functions of *BcSHO1* and *BcSLN1* in *B. cinerea*, we generated both single and double deletion mutants Δ*BcSho1*, Δ*BcSln1*, and Δ*BcSln1-Sho1*, using homologous recombination strategy (Supplementary Figures S1A,C). The single deletion mutants were verified by Southern blotting analysis (Supplementary Figures S1B,D). To confirm that the phenotypic changes of mutants were due to gene deletion, the mutants were complemented with the full-length of *BcSHO1* or *BcSLN1*, respectively, and generated the complemented strains Δ*BcSho1-C* and Δ*BcSln1-C*.

### *BcSHO1* and *BcSLN1* Are Involved in Vegetative Growth and Conidiation

**FIGURE 2:**
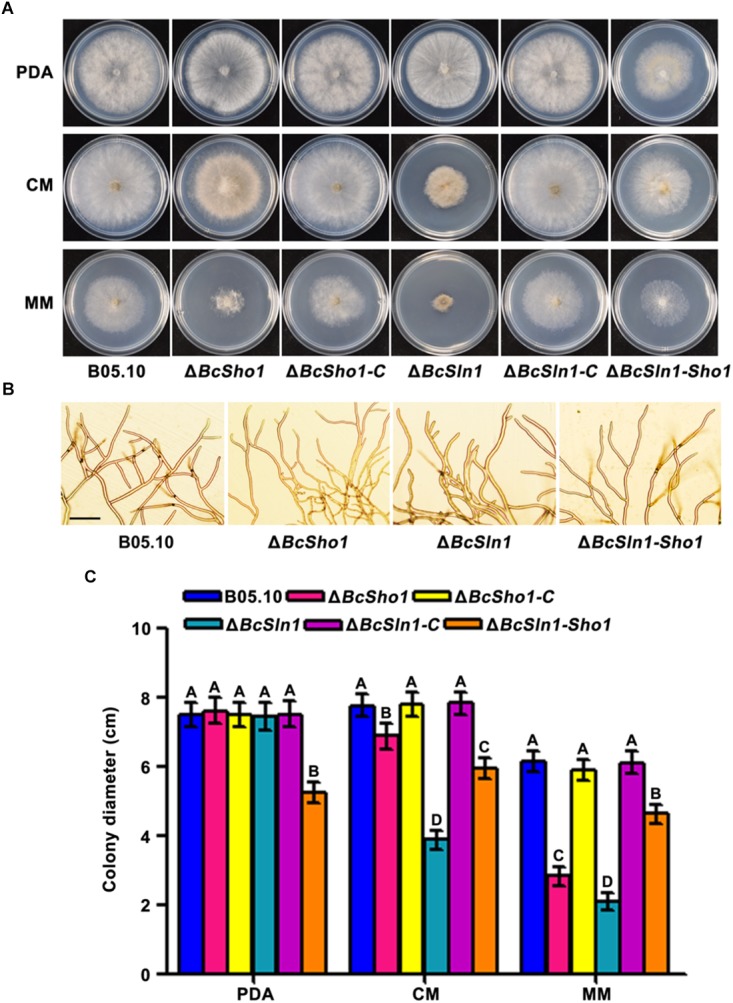
Roles of *BcSHO1* and *BcSLN1* in vegetative growth. **(A)** Colony morphology of the wild-type B05.10, the single deletion mutants Δ*BcSho1* and Δ*BcSln1*, the double deletion mutant Δ*BcSln1-Sho1*, and the complemented mutants Δ*BcSho1-C* and Δ*BcSln1-C* on PDA, CM and MM media at 25°C for 4 days. **(B)** Hyphal morphology of each strain growing on PDA plates. Bar, 100 μm **(C)** Colony diameters were measured in each independent biological experiment and statistically analyzed by Duncan analysis. Bars represent standard errors from three replications. Values on the bars followed by the same letter are not significantly different at *P* = 0.05.

Δ*BcSho1* and Δ*BcSln1* showed similar growth rates to the WT strain B05.10 on PDA, but grew slower than B05.10 on either CM or MM especially Δ*BcSln1*. Δ*BcSln1-Sho1* showed significantly reduced radial growth on all media tested compared with that of B05.10 (Figure 2A,C). Moreover, Δ*BcSho1* and Δ*BcSln1* formed denser colonies, in contrast, Δ*BcSln1-Sho1* formed sparser colony with reduced aerial hyphae (Figure 2A). Microscopic examination showed that the hyphae of Δ*BcSho1* and Δ*BcSln1* beared more short lateral branches especially Δ*BcSln1* produced shorter hyphae (Figure 2B).

**FIGURE 3:**
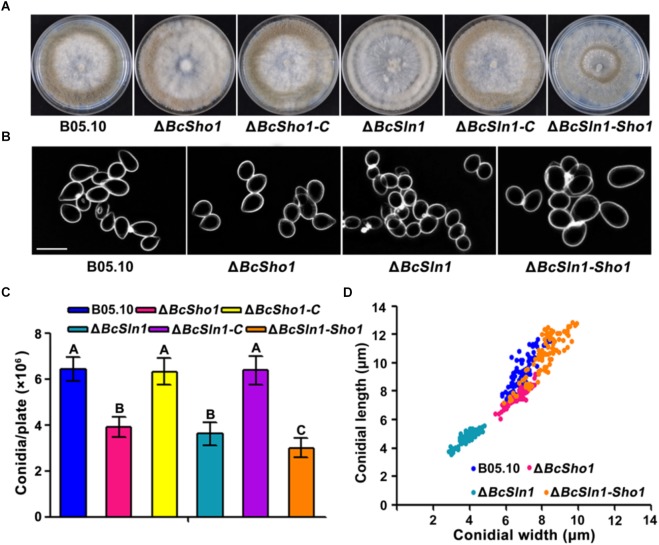
Roles of *BcSHO1* and *BcSLN1* in conidiation. **(A)** Comparison of conidiation among B05.10, Δ*BcSho1*, Δ*BcSln1*, and Δ*BcSln1-Sho1* after 10 days incubation on PDA plates at 20°C under white light. **(B)** Conidial morphology of the each strain. Scale bar 10 μm. **(C)** The number of conidia produced by each strain. Bars represent standard errors from three replications. Values on the bars followed by the same letter are not significantly different at *P* = 0.05. **(D)** Conidial size of the each strain. More than 100 conidia were measured under a microscope in each experiment.

Conidia serve as the primary inoculum for disease cycle of *B. cinerea* (Williamson et al., 2007). After incubated on PDA for 10 days, the amounts of conidia produced by Δ*BcSho1*, Δ*BcSln1*, and Δ*BcSln1-Sho1* were significantly less than that of B05.10 (Figure 3A,C). Moreover, the conidia of Δ*BcSho1*, Δ*BcSln1*, and Δ*BcSln1-Sho1* showed morphological abnormality and shifts in size (Figure 3B,D). These results indicate that both *BcSHO1* and *BcSLN1* play important roles in vegetative growth and conidiation of *B. cinerea*.

### *BcSHO1* and *BcSLN1* Are Involved in Sclerotial Formation, Morphogenesis and Germination

**FIGURE 4:**
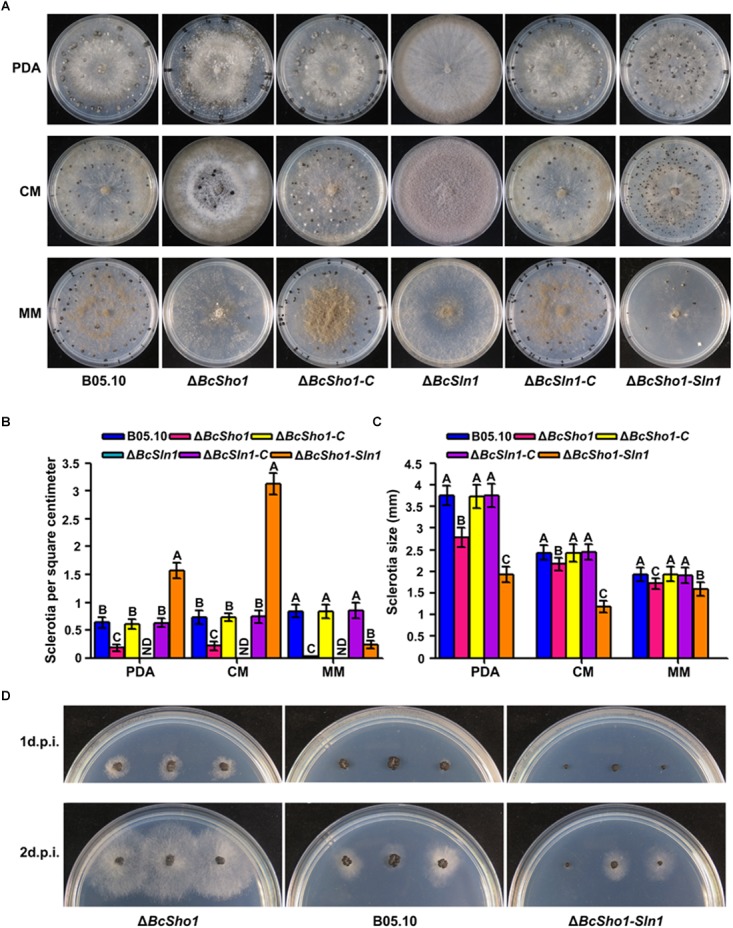
Roles of *BcSHO1* and *BcSLN1* in sclerotial formation. **(A)** Colony morphology of sclerotial formation. B05.10, Δ*BcSho1*, Δ*BcSln1*, Δ*BcSln1-Sho1*, Δ*BcSho1-C*, and Δ*BcSln1-C* were cultured on PDA, CM and MM media at 4°C for 4 weeks in darkness. **(B)** The number of sclerotia produced by each strain on PDA, CM, and MM media. ND, not detected. Bars represent standard errors from three replications. Values on the bars followed by the same letter are not significantly different at *P* = 0.05. **(C)** The size of sclerotia produced by each strain. Bars represent standard errors from three replications. Values on the bars followed by the same letter are not significantly different at *P* = 0.05. **(D)** Sclerotial germination of each strain on PDA plates at 25°C at 1 and 2 days post-incubation (d.p.i.).

Sclerotia represent an important survival mechanism in *B. cinerea* (Williamson et al., 2007), we therefore investigated the impacts of *BcSHO1* and *BcSLN1* on sclerotial formation. After incubation for 4 weeks, B05.10, Δ*BcSho1*, and Δ*BcSln1-Sho1* formed sclerotia on all tested media, even though the size of sclerotia produced by Δ*BcSho1* and Δ*BcSln1-Sho1* were smaller than that of B05.10. Moreover, Δ*BcSho1* produced less sclerotia on all tested media, and Δ*BcSln1-Sho1* produced more sclerotia on PDA and CM, but less sclerotia on MM than that of B05.10. Notably, Δ*BcSln1* failed to develop any sclerotia on all tested media, but produced a large number of conidia (Figure 4A–C), suggesting that *BcSLN1* as such is not required for conidiation, but for its correct regulation under light vs. dark conditions. In addition, the sclerotial germination of Δ*BcSho1* and Δ*BcSln1-Sho1* was severely delayed compared with that of B05.10 (Figure 4D). These results indicate that *BcSLN1* is essential for sclerotial development in *B. cinerea*, while *BcSHO1* is less important, and there exists cooperative regulation between them.

### *BcSHO1* and *BcSLN1* Are Redundantly Required for Virulence

**FIGURE 5:**
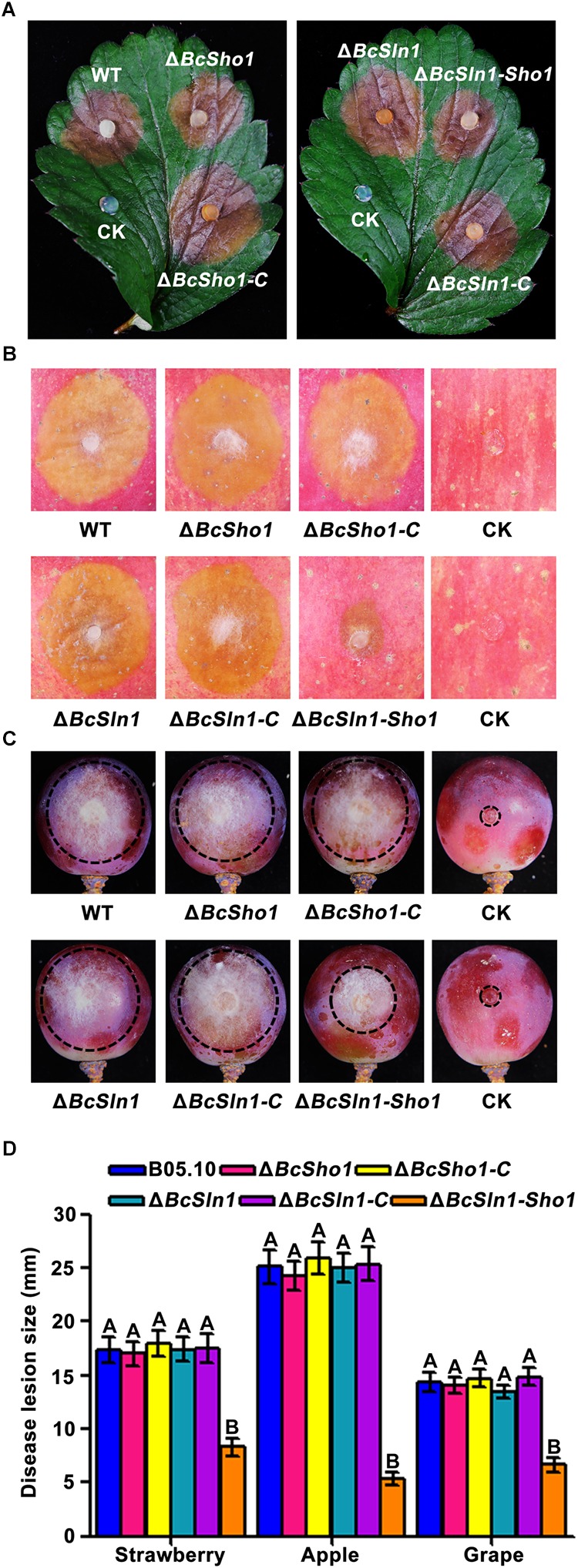
Roles of *BcSHO1* and *BcSLN1* in virulence. Infection tests on different plant tissues following incubation with B05.10, Δ*BcSho1*, Δ*BcSln1*, Δ*BcSln1-Sho1*, Δ*BcSho1-C*, and Δ*BcSln1-C*. Agar plugs without fungal mycelia were used as negative controls (CK). **(A)** Disease symptom on strawberry leaves at 3 days post-incubation (d.p.i.). **(B)** Disease symptom on apple fruits at 4 d.p.i. **(C)** Disease symptom on grape fruits at 4 d.p.i. **(D)** The diameter of disease lesions caused by each strain. Bars represent standard errors from three replications. Values on the bars followed by the same letter are not significantly different at P = 0.05.

To determine the roles of *BcSHO1* and *BcSLN1* in virulence, infection tests were performed on different plant tissues. As shown in Figure 5, both Δ*BcSho1* and Δ*BcSln1* showed similar pathogenicity to the WT strain B05.10 on strawberry leaves and apple and grape fruits, however, the virulence of Δ*BcSln1-Sho1* reduced significantly, suggesting that there is functional redundancy between *BcSHO1* and *BcSLN1* in terms of pathogenesis.

### *BcSHO1* and *BcSLN1* Redundantly Regulate Osmotic Stress Tolerance

**FIGURE 6:**
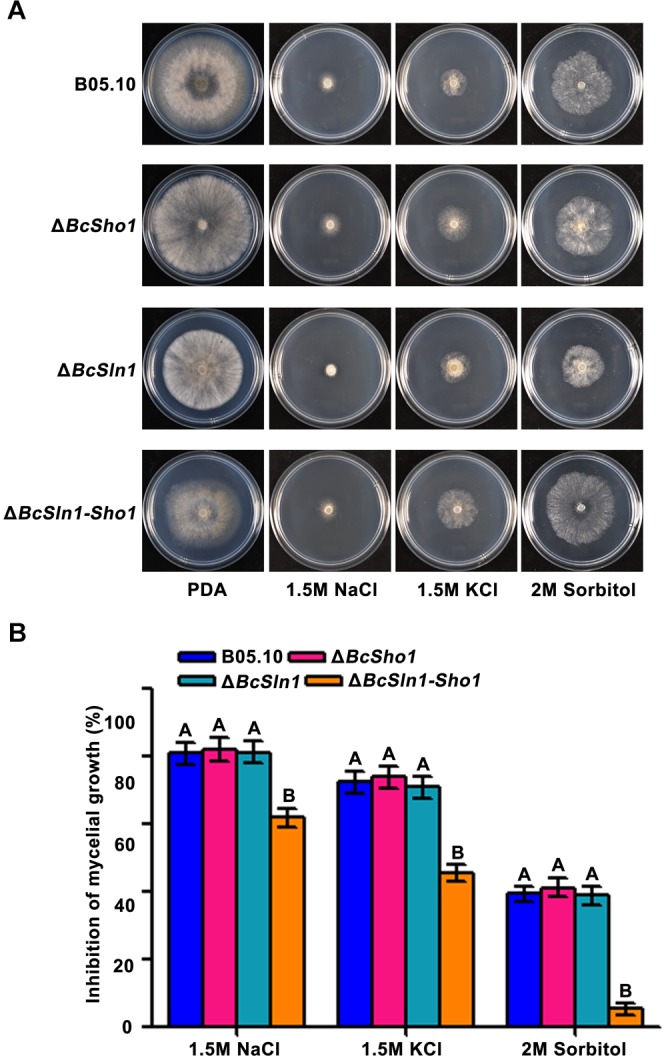
Sensitivity of B05.10, Δ*BcSho1*, Δ*BcSln1*, and Δ*BcSln1-Sho1* to osmotic stress. **(A)** All strains were grown on PDA plates amended with NaCl, KCl, or D-sorbitol at the indicated concentrations at 25°C for 4 days. **(B)** Statistical analysis of the growth inhibition rate of all strains under osmotic stress. Bars represent standard errors from three replications. Values on the bars followed by the same letter are not significantly different at *P* = 0.05.

Sho1 and Sln1 serve as the osmosensors of HOG pathway in yeast (Rispail et al., 2009), so we were interested in the impacts of *BcSHO1* and *BcSLN1* on osmotic stress tolerance in *B. cinerea*. As shown in Figure 6, Δ*BcSln1-Sho1* showed significantly reduced sensitivity to osmotic stress generated by NaCl, KCl or sorbitol compared with that of B05.10, however, the sensitivity of Δ*BcSho1* and Δ*BcSln1* to osmotic stress remained unchanged.

Since phenylpyrrole and dicarboximide fungicides have been reported to activate HOG pathway in some fungal pathogens (Kojima et al., 2004), we tested the sensitivity of Δ*BcSho1*, Δ*BcSln1*, and Δ*BcSln1-Sho1* to the phenylpyrrole fungicide, fludioxonil and the dicarboximide fungicides, procymidone and iprodione. Δ*BcSho1* showed slightly reduced sensitivity (two-fold) to all tested fungicides, and Δ*BcSln1-Sho1* only showed reduced sensitivity to fludioxonil, whereas the sensitivity of Δ*BcSln1* to all tested fungicides remained unchanged (Table 1). These results indicate that *BcSHO1* and *BcSLN1* play redundant roles in osmotic stress response in *B. cinerea*.

**Table 1:** Sensitivity of the wild-type strain B05.10, the *BcSHO1* deletion mutant Δ*BcSho1*, the *BcSLN1* deletion mutant Δ*BcSln1*, and the *BcSHO1* and *BcSLN1* double deletion mutant Δ*BcSln1-Sho1* to different fungicides.

Strain		EC_50_ (μg/ml)^∗^	
	**Fludioxonil**	**Procymidone**	**Iprodione**
B05.10	0.0137a	0.2379a	0.2832a
Δ*BcSho1*	0.0284b	0.3852b	0.4532b
Δ*BcSln1*	0.0115a	0.2217a	0.2589a
Δ*BcSln1-Sho1*	0.0252b	0.2054a	0.2642a


*^∗^Means in the columns followed by the same letter are not significantly different according to the Fisher’s least significant difference (LSD) test at P = 0.05.*

### *BcSHO1* and *BcSLN1* Function Redundantly to Negatively Regulate Glycerol Production and Hog1 Phosphorylation

**FIGURE 7:**
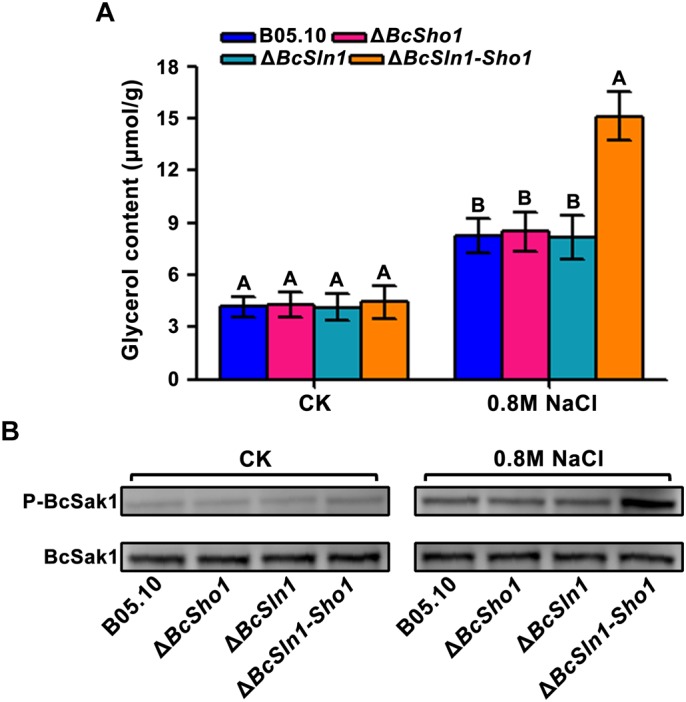
Glycerol content and BcSak1 phosphorylation of B05.10, Δ*BcSho1*, Δ*BcSln1*, and Δ*BcSln1-Sho1*. **(A)** The amounts of glycerol (per g mycelial dry weight) produced by each strain in YEPD medium amended with 0.8 M NaCl. The cultures without any treatment were used as control (CK). Bars represent standard errors from three replications. Values on the bars followed by the same letter are not significantly different at *P* = 0.05. **(B)** The BcSak1 and phosphorylated BcSak1 proteins were detected using the yeast anti-Hog1p (C-terminal anti-Hog1) and phosphorylated p38 (Thr180/Tyr182) antibodies, respectively.

The HOG pathway maintains osmotic homeostasis by Hog1 phosphorylation which regulates glycerol production (Burg et al., 1996), we therefore examined glycerol content and the phosphorylation of BcSak1 (yeast Hog1). Under normal conditions, the glycerol contents of Δ*BcSho1*, Δ*BcSln1*, and Δ*BcSln1-Sho1* were similar to that of B05.10, but when treated with NaCl, Δ*BcSln1-Sho1* showed significantly high glycerol production (Figure 7A).

Phosphorylation analysis revealed that the phosphorylation level of BcSak1 in Δ*BcSln1-Sho1* was significantly increased under hyperosmotic conditions compared with that of B05.10, Δ*BcSho1* and Δ*BcSln1* (Figure 7B), which was consistent with the above results. These results indicate that *BcSHO1* share redundancy with *BcSLN1* in regulating the response of *B. cinerea* to external osmotic stress.

## Discussion

Living cells are equipped with precise mechanisms for sensing external signal, such as osmosis, oxidation, and hormones, which allow adaptation to environment through a variety of cellular responses triggered by sensing and subsequent signaling systems (Bahn et al., 2007). In this study, we characterized the roles of two sensor proteins of HOG pathway in *B. cinerea* and found that the single or double deletion mutants of *BcSHO1* and *BcSLN1* exhibited severe defects in growth, conidium and sclerotium formation, however, only the *BcSHO1* and *BcSLN1* double deletion mutant showed a significant reduction in virulence. In addition, single or double deletion of *BcSHO1* and *BcSLN1* made no difference to oxidative and cell wall stress sensitivity (data not shown), and only the double deletion increased the phosphorylation level of BcSak1 (an ortholog of *S. cerevisiae* Hog1), which in return resulted in tolerance to osmotic stresses. In *S. cerevisiae*, the Sho1 and Sln1 branches are redundant for promoting growth on high-osmolarity condition, but they also display some functional specificities (O’Rourke and Herskowitz, 2003).

Both Sho1 and Sln1 proteins in budding yeast serve as biosensors to response to extracellular environmental stimuli (O’Rourke et al., 2002). In this study, compared with the WT strain, single deletion of *BcSHO1* or *BcSLN1* in *B. cinerea* exhibited similar sensitivity to osmotic and oxidative stress and virulence suggesting that the roles of *BcSHO1* and *BcSLN1* are dramatically different from that of *S. cerevisiae*. Likewise, as the major sensing proteins in pathogenic fungi, the hybrid histidine-kinase (HK) of class III are generally implicated in oxidant condition adaptation, dicarboximide and phenylpyrrole antifungal sensitivity, cell wall integrity, and conidiation, however, it still remains the species-specific roles (Defosse et al., 2015; Hérivaux et al., 2016).

Pathogenicity experiment revealed that the double deletion mutant of *BcSHO1* and *BcSLN1* caused the decreased virulence, suggesting that BcSho1 and BcSln1 have an additive effect on pathogenicity of *B. cinerea*. However, the disease severity of Δ*BcSln1-Sho1* was dramatically less than that of Δ*FgSln1-Sho1* in *F. graminearum* (Gu et al., 2014), indicating that the involvement of the BcSln1 and BcSho1 branches in pathogenesis is less important than that in *F. graminearum*.

Previous study in *F. graminearum* showed that disruption of *FgSHO1* results in a reduction in sporulation, whereas the deletion of *FgSLN1* displays an undetectable change in sporulation (Gu et al., 2014). MoSho1 and MoSln1 are required for sporulation in *M. oryzae* (Zhang et al., 2010; Liu et al., 2011), and deletion of tcsA (a homolog of Sln1 in *S. cerevisiae*) attenuated sporulation in *A. nidulans* (Furukawa et al., 2002). In the current study, both *BcSHO1* and *BcSLN1* showed dramatically decrease in sporulation, suggesting that the effect of Sln1 on sporulation differs in fungi. In addition, the spore sizes from the mutants and WT strain differed considerably. Spores in statistical size from smallest to biggest were ranked as an order of Δ*BcSln1* < Δ*BcSho1* < WT strain (B05.10) < Δ*BcSln1-Sho1*. To our knowledge, this is first reported that the roles of *BcSHO1* and *BcSLN1* are involved in the size of spore.

Sclerotia are asexual and resting structures of some agricultural crop pathogens, including *Claviceps purpurea*, *Sclerotinia sclerotiorum*, and *B. cinerea* (Coley-Smith and Cooke, 1971). Disruption of BcSak1 in *B. cinerea* causes increased number of sclerotia (Segmüller et al., 2007). In this study, Δ*BcSln1*, not Δ*BcSho1*, failed to form sclerotia, while the double deletion of *BcSHO1* and *BcSLN1* resulted in increased sclerotial development on rich medium. Notably, Δ*BcSln1* produced a large number of conidia instead of sclerotia in the darkness. For *Botrytis*, vegetative mycelia differentiate conidiophores and conidia when incubated in the light and sclerotia in constant darkness (Schumacher, 2017). Therefore, *BcSLN1* participates in the light-dependent development of *B. cinerea*. Moreover, the procedure of sclerotia formation mainly includes initiation, development and maturation, and three groups of factors are reportedly involved in sclerotial formation, including environmental signals, Ser/Thr phosphatase type 2A and 2B, cellular sensing and signal transduction (Giesbert et al., 2011). BcSho1 and BcSln1 branches may play a role in sensing and signal transduction for sclerotial formation in *B. cinerea*.

Besides osmotic stress, HOG pathway is also involved in cellular responses to oxidative, ultraviolet radiation, heavy metal, and heat stress in fungi (Irniger et al., 2016). In *Aspergillus nidulans*, the SakA (yeast Hog1) mutants showed more sensitive to osmotic and oxidative stress, and the phosphorylated SakA undergoes nuclear import under osmotic and oxidative stress conditions (Jaimes-Arroyo et al., 2015). In *Neurospora crassa*, the Os2 (yeast Hog1) mutants are highly sensitive to high osmolarity and exhibit resistance to fungicides such as phenylpyrrole (Zhang et al., 2002). The FgOS-2 deletion mutants are sensitive to osmotic stress but not to oxidative stress or fungicides in *F. graminearum* (Nguyen et al., 2012). In this study, the double-gene deletion mutant Δ*BcSln1-Sho1* exhibited increased tolerance to osmotic stress by BcSak1 phosphorylation, indicating that an extra pathway may be triggered and/or strengthened to activate the HOG pathway. In *S. cerevisiae*, the HOG pathway perceives the osmotic stress signals from the five transmembrane osmosensors, including Sho1, Sln1, Msb1, Hkr1, and Opy2 (Maeda et al., 1994). Opy2 reportedly serves as a third osmosensor in the Sho1 branch that anchors ste50 and ste11 in budding yeast (Yamamoto et al., 2010). According to the data from an online platform of KEGG^3^, *B. cinerea* MAPK-HOG pathway harbors three transmembrane osmosensors, i.e., BcSln1, BcSho1 and BcOpy2 (Gene code: BCIN_04g03130 in *B. cinerea* B05.10). We therefore postulated that the MAPK-HOG branch [Opy2-Ste50-Ste11-Pbs2-Sak1(Hog1)] is triggered at least on the condition of the double disruption of *BcSHO1* and *BcSLN1* in *B. cinerea*. Additionally, only Δ*BcSho1* displayed stable resistance against phenylpyrrole and dicarboximide fungicides tested, while deletion of Sln1, not Sho1 in *C. neoformans*, *A. nidulans*, and *A. fumigatus* exhibited resistance against phenylpyrrole or/and dicarboximide fungicides (Chen et al., 2012), suggesting that the unique effect of *BcSHO1* on the resistance against these fungicides maybe results from the specific branch [Opy2-Ste50-Ste11-Pbs2-Sak1(Hog1)]. Detailed characterization of this branch and their orchestration with other MAPK-HOG signaling cascades is under scrutiny.

## Author Contributions

WR, CC, and QG conceived and designed the experiments. WR, NL, and YY performed the experiments, analyzed the data, prepared figures and tables. WR, QY, and CC wrote and revised the paper. All authors have read and approved the final manuscript.

## Conflict of Interest Statement

The authors declare that the research was conducted in the absence of any commercial or financial relationships that could be construed as a potential conflict of interest.

## Footnotes

1 http://fungi.ensembl.org/Botrytis_cinerea/Info/Index

2 http://smart.embl-heidelberg.de/

3 https://www.kegg.jp/

## Supplementary Material

FIGURE S1 Generation and identification of *BcSHO1* and *BcSLN1* deletion mutants. **(A)** Gene replacement strategy of *BcSHO1*. **(B)** Southern blotting analysis of *BcSHO1* mutant. **(C)** Gene replacement strategy of *BcSLN1*. **(D)** Southern blotting analysis of *BcSLN1* mutant.

TABLE S1 Oligonucleotide primers used in this study.
