# Supplementary figures and images for: The Sensor Proteins BcSho1 and BcSln1 Are Involved in, Though Not Essential to, Vegetative Differentiation, Pathogenicity and Osmotic Stress Tolerance in Botrytis cinerea
Source: Front Microbiol. 2019 Feb 25;10:328. doi: 10.3389/fmicb.2019.00328 (PMC6397835; doi:10.3389/fmicb.2019.00328)

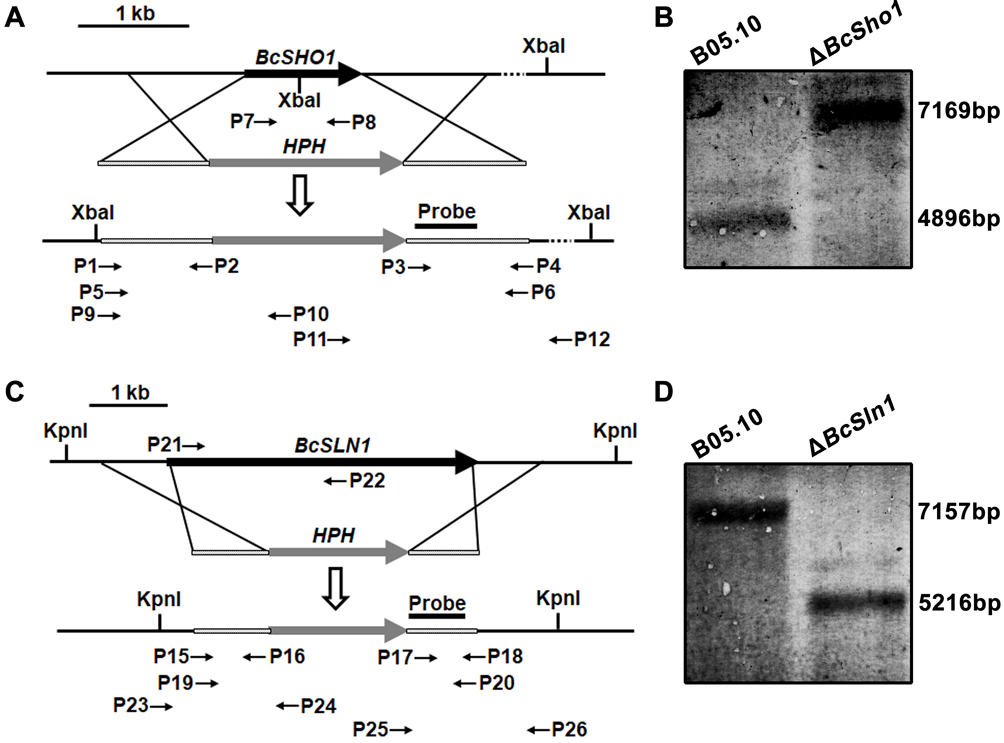

Supplement: FIGURE S1 — Generation and identification of BcSHO1 and BcSLN1 deletion mutants. (A) Gene replacement strategy of BcSHO1. (B) Southern blotting analysis of BcSHO1 mutant. (C) Gene replacement strategy of BcSLN1. (D) Southern blotting analysis of BcSLN1 mutant. [file Image_1.TIF]
