# Supplementary material for: The Sensor Proteins BcSho1 and BcSln1 Are Involved in, Though Not Essential to, Vegetative Differentiation, Pathogenicity and Osmotic Stress Tolerance in Botrytis cinerea
Source: Front Microbiol. 2019 Feb 25;10:328. doi: 10.3389/fmicb.2019.00328 (PMC6397835; doi:10.3389/fmicb.2019.00328)
Supplement: TABLE S1 — Oligonucleotide primers used in this study. [file Table_1.DOC]

**Table S1**. Oligonucleotide primers used in this study.

| Primer | Sequence (5’-3’) | Relevant characteristics |
| --- | --- | --- |
| P1 | TTCCGTTATCCGTTGTCCG | PCR primers to amplify *BcSHO1* upstream fragment for construction of the gene deletion vector |
| P2 | CCACCAGCCAGCCAACAGCT  CCCCTTGCGTCGTGAGGGGGA |
|  |  |  |
| P3 | CAATACGCAAACCGCCTCTCC  CCGGATGAATCAGGTTCTTGGA | PCR primers to amplify *BcSHO1* downstream fragment for construction of the gene deletion vector  PCR primers to amplify the hygromycin B B (*HPH*) gene  PCR primers to amplify the deletion vector of *BcSHO1* with the double-joint PCR products as template  PCR primers to identificate the *BcSHO1* fragment  PCR primers to identificate the integration of *HPH* at the left junction of *BcSHO1*  PCR primers to identificate the integration of *HPH* at the right junction of *BcSHO1*  PCR primers to amplify the probe fragment for *BcSHO1* Southern blotting  PCR primers to amplify *BcSLN1* upstream fragment for construction of the gene deletion vector  PCR primers to amplify *BcSLN1* downstream fragment for construction of the gene deletion vector  PCR primers to amplify the deletion vector of *BcSLN1* with the double-joint PCR products as template  PCR primers to identificate the *BcSLN1* fragment  PCR primers to identificate the integration of *HPH* at the left junction of *BcSLN1*  PCR primers to identificate the integration of *HPH* at the right junction of *BcSLN1*  PCR primers to amplify the probe fragment for *BcSLN1* Southern blotting  PCR primers to amplify *BcSHO1* upstream fragment for construction of the gene deletion vector  PCR primers to amplify *BcSHO1* downstream fragment for construction of the gene deletion vector  PCR primers to amplify the geneticin B (*NEO*) gene  PCR primers to identificate the integration of *NEO* at the left junction of *BcSHO1*  PCR primers to identificate the integration of *NEO* at the right junction of *BcSHO1*  PCR primers to amplify the full *BcSHO1* fragment used for construction of the BcSho1-GFP vector  PCR primers for the identification of the in-frame BcSho1-GFP fusion vector  PCR primers to amplify the full *BcSLN1* fragment used for construction of the BcSln1-GFP vector  PCR primers for the identification of the in-frame BcSln1-GFP fusion vector |
| P4  HPH-F  HPH-R  P5  P6  P7  P8  P9  P10  P11  P12  P13  P14  P15  P16  P17  P18  P19  P20  P21  P22  P23  P24  P25  P26  P27  P28  P29  P30  P31  P32  G418-F  G418-R  P33  P34  P35  P36  P37  P38  P39  P40  P41  P42  P43  P44 | ACCAATCCCCTCCTCTTCCT  GGGAGCTGTTGGCTGGCT  GGGGAGAGGCGGTTTGCG  CCCAGGACCGTTACCACT  CCTCCCTCTTCTCATTTCA  GAATGCGTGGAATGAGTATGG  TTGGATGATTTCTCCTGGTGT  GCTTTCCGTTATCCGTTGT  TGGAGCGAGGCGATGTTC  GAACCCGCTCGTCTGGCTAAG  CCCACTATCTGGCATTCCTCC  GGGGATGGGATTAGTTTCT  TGTCCGCATCATTATCTGT  ATGTCCATCACCCCTTGC  CCACCAGCCAGCCAACAGCT  CCCCTCCAGACCGTTGTATGT  CAATACGCAAACCGCCTCTCC  CCCGACCAGCTCTCAAGCAAG  AGATAAATCGCCAGGTGCTC  TGATAAGCCCAGACGAGACC  CCATTTCACCATTCCACCAT  AGCCTTACGGCATCACTCA  AATACCGCCTTCGTCCACT  GGATGTTGTGGCTGATGG  ACTCGCCGATAGTGGAAA  TCCTCGTTCCTGTCTGCTAA  CAATCAACTTCCACCTCTAATC  CTTCACCTCCATCACCTTTAA  AACCATTTCACCATTCCACCA  TTCCGTTATCCGTTGTCCG  CAATATCATCTTCTGTCGA  CCTTGCGTCGTGAGGGGGA  TTCTTGACGAGTTCTTCTGA  GGATGAATCAGGTTCTTGGA  GTCGACAGAAGATGATATTG  TCAGAAGAACTCGTCAAGAA  GCCATGCGTACTGCTTA  CGATTGTCTGTTGTGCC  GGGCACAACAGACAATC  CTCCCTTTCCACTACTCAT  CCATCACATCACAATCGATCCAACCATGAACCAATCGGATTATAAAGGTC  TACTTACCTCACCCTTGGAAACCATTAACAAAATTAGGTAATTCGAAGGC  CAATCTTGGACCAAATGG  CCTCTGGCATTGCAGACTTG  CCATCACATCACAATCGATCC  AACCATGAGGATTGGTATCAGG  TACTTACCTCACCCTTGGAAACC  ATTGTAGAGGTTAAAGGTGATG  GCTATGAGTCCGGAATGG  CCTCTGGCATTGCAGACTTG |
